# The Norwegian version of the nursing student mentors’ competence instrument (MCI): A psychometric validation study

**DOI:** 10.1016/j.ijnsa.2024.100183

**Published:** 2024-02-03

**Authors:** Silje Christin Wang Linnerud, Camilla Olaussen, Jaroslav Zlamal, Monica Evelyn Kvande, Kristine Haddeland, Andréa Aparecida Goncalves Nes

**Affiliations:** a Lovisenberg Diaconal University College, Norway; b Department of Anaesthesiology and Surgery, University Hospital of North Norway, Tromsø, Norway; c University of Agder, Norway; d University of Stavanger, Norway

**Keywords:** Clinical practice, Competence, Instrument validation, Mentoring, Nursing education, Psychometrics

## Abstract

**Background:**

Nurse mentors require competence to mentor nursing students in clinical practice, including specific knowledge and skills. Evaluating mentor competence is crucial in developing and ensuring the high-quality mentoring of nursing students. The nursing student mentors’ competence instrument is one of the few valid instruments for assessing the competence of nurses as mentors.

**Objectives:**

To translate the nursing student mentors’ competence instrument into Norwegian and evaluate its psychometric properties.

**Design:**

The research employed a cross-sectional study design.

**Settings:**

Data were collected from nurse mentors at nursing homes, hospitals, home nursing care and mental health care units in Norway from 2021 to 2022.

**Participants:**

A total of 458 registered nurses with experience of mentoring nursing students participated in the study, of which data was used to conduct psychometric testing.

**Methods:**

The nursing student mentors’ competence instrument was translated and evaluated in six steps: Forward translation, forward translation synthesis, backward translation, backward translation synthesis, cognitive debriefing and psychometric testing. The validity and reliability of the translated instrument were investigated using confirmatory factor analysis (CFA) and Cronbach's alpha.

**Results:**

The translated instrument showed acceptability. The CFA goodness-of-fit indices showed acceptable model fit (χ^2^/df = 2.547, SRMR = 0.051, CFI = 0.919, RMSEA = 0.058), and the Cronbach's alpha values for the instrument's subscales ranged from 0.77 to 0.95.

**Conclusions:**

The Norwegian version of the nursing student mentors’ competence instrument shows potential as a useful instrument for assessing current and required competencies of nurse mentors in clinical practice in nursing education.

**What is already known about the topic?**

• Adequate mentorship plays a crucial role in enhancing the clinical learning experience of nursing students.
• There is a lack of available instruments for assessing nurses’ current and required competence for mentoring nursing students.

Alt-text: Unlabelled box

**What this paper adds**

• The psychometric testing in this study demonstrates sufficient validity and reliability of the nursing student mentors’ competence instrument (MCI) in a Norwegian context.
• The MCI has the potential to assess Norwegian nursing mentors’ mentoring competence.
• Educational institutions can utilize the MCI to support the development of mentor competence in healthcare settings where nursing students are placed.

Alt-text: Unlabelled box

## 1 Introduction

Clinical practice is an essential part of nursing education programmes worldwide (Baker et al., 2021). In 30 European countries, including Norway, educational institutions are obliged to follow the Bologna process, which prescribes that nursing students must spend half their nursing education programme in clinical practice (Collins and Hewer, 2014; European Council, 2013). In Norway, the bachelor's degree in nursing is a three-year programme that follows a European Union (EU) directive under which clinical practice is mandatory and is mentored in various areas of the health care service (EU Directive 2013/55/EU). Mentoring is defined by Mikkonen (2017, p. 28) as the ‘facilitation of students’ learning by a mentor who is creating an open and safe learning environment for the students. It also includes guiding students through their learning process and strengthening the students’ professionalism’. The mentoring is usually performed by registered nurses employed at the health care institutions and departments where the nursing students perform their clinical practice (Bjørk et al., 2014; Mortjernet et al., 2022). Although Norway follows several mentoring models (Koch et al., 2023), most nursing students are mentored by onsite nurse mentors during their clinical practice as is very common in other European countries (Mikkonen et al., 2020; Nes et al., 2020).

Research shows that variation in nurse mentors’ competence directly affects nursing students’ learning outcomes in clinical practice placements (Myrick and Yonge, 2001; Tang, 2021). Nurse mentors must be competent in mentoring, demonstrating specific knowledge of students’ learning objectives and the core elements of nursing and learning processes as well as possessing skills linked to personal qualities and the ability to build trusting reciprocal relationships, to cooperate with stakeholders and to provide feedback and evaluation (Mikkonen et al., 2022). These professional, pedagogical and personal skills play a significant role in supporting nursing students’ learning process (Ford et al., 2016; Gurková et al., 2016; Tuomikoski et al., 2018) and help students become confident professionals (Mikkonen et al., 2020; Tuomikoski et al., 2018, 2020). Positive mentoring results greatly depend on the availability of nurse mentors who are willing to take on their mentoring responsibilities (Sedgwick and Harris, 2012), but nurse mentors may face challenges—such as difficulty in balancing patient care and student mentoring (Jayasekara et al., 2018) and limited mentoring competence and experience, that may diminish student outcomes in clinical practice placements (Aigeltinger et al., 2012; Jonsén et al., 2013). To strengthen the development of nurse mentors’ competence, the EU competence guideline highlights following areas: (1) nurse mentors’ individual competencies, (2) interactions and resources in the workplace, (3) cultural competence and (4) competence in supporting students’ learning process (Oikarainen et al., 2021). Other elements may also be important for success in mentoring nursing students. For example, an Australian study found that the ideal qualities related to clinical mentoring were role modelling by nurse mentors and a combination of students working one-on-one with the nurse mentor and working together in groups during clinical practice placements (King et al., 2020).

The evaluation of mentoring competence is crucial to the development and enhancement of competence and to ensuring the high-quality mentoring of nursing students during clinical practice placements (Tuomikoski et al., 2018). Valid instruments for assessing the competence of nurses as mentors are few, but the nursing student mentors’ competence instrument (MCI) is designed to extensively assess mentoring competence in clinical practice (Tuomikoski et al., 2018). The MCI is a self-report instrument of a registered nurse's mentoring competence. The MCI was originally developed in Finland and is used among nurse mentors to assess current and required competence for mentoring nursing students. The MCI asks nurse mentors about their perceptions in areas such as mentoring practices in the workplace, the mentor's characteristics, the mentor's motivation, goal orientation in mentoring, reflection during mentoring, student-centred evaluation and constructive feedback (Mikkonen et al., 2020). Data within such areas is important in evaluating whether mentors possess the required competence to mentor nursing students or if strategies for strengthening mentoring competence is needed. Currently, the MCI has been translated and validated in several European countries (Mikkonen et al., 2020; Tuomikoski et al., 2020). The present study was inspired by the need for a valid instrument to identify and assess areas of mentoring competence in clinical practice that may require strategies for improvement in Norwegian nursing education. Therefore, the study aimed to translate the MCI into Norwegian and evaluate its psychometric properties. The research question was: ‘How is the validity and reliability of the nursing student mentors’ competence instrument (MCI) in a Norwegian context?’

## 2 Methods

### 2.1 Design

This study had a cross-sectional design, including translating the MCI from English to Norwegian and testing its psychometric properties.

### 2.2 Setting and participants

The researchers invited nurse mentors at Norwegian nursing homes, hospitals, home nursing care and mental health care units who had experience of mentoring nursing students in bachelor's or master's programmes to participate in the study (psychometric testing). The participants provided informed consent. These four specified health care units are frequently chosen as clinical practice placements in Norway. However, nursing students have the flexibility to engage in clinical practice placements in diverse health care institutions where registered nurses are employed. Throughout the three-year educational programme, during six clinical practice periods varying from 8 to 12 weeks, undergraduate nursing students receive daily mentoring from a registered nurse. Within a 30-hours weekly commitment to clinical practice placement, students also benefit from regular mentoring sessions provided by registered nurses (Bjørk et al., 2014).

### 2.3 Instrument

The original MCI was a 10-factor model comprising 63 items designed to assess the 10 factors (Tuomikoski et al., 2018). Recently, however, the MCI was revised and evaluated in an international cross-sectional survey coordinated in five European countries, which resulted in a seven-factor MCI model with a total of 43 corresponding items (Mikkonen et al., 2020). The Cronbach's alpha subscale values in the seven-factor model ranged between 0.83 and 0.94, the overall variance in an exploratory factor analysis was 68 % and the seven-factor MCI showed acceptable goodness-of-fit indices in a confirmatory factor analysis (CFA) (Mikkonen et al., 2020). This study used the seven-factor MCI, which includes the following factors (subscales) with corresponding items as shown in Table 1: I. mentoring practices in the workplace (six items), II. mentor's characteristics (seven items), III. mentor's motivation (five items), IV. goal-oriented mentoring (six items), V. reflection during mentoring (six items), VI. student-centred evaluation (nine items) and VII. constructive feedback (four items) (Mikkonen et al., 2020).

**Table 1:** The MCI seven-factor model and corresponding items (Mikkonen et al., 2020).

Factors/subscales	Items
I. Mentoring practices in the workplace	I.1. I am well-acquainted with the quality requirements and criteria relating to clinical practice and learning at work in social and health care.
	I.2. I am well-acquainted with the mentoring process of students in clinical practice within my organization.
	I.3. I am aware of generally agreed practices for student mentoring within my organization.
	I.4. I follow generally agreed practices during student mentoring.
	I.5. I am familiar with the tasks and responsibilities of the person in charge of mentoring students.
	I.6. I am familiar with the tasks and responsibilities of the mentor.
II. Mentor's characteristics	II.1. It is easy for students to approach me.
	II.2. I am empathetic towards students during mentoring.
	II.3. I am flexible during the mentoring of students.
	II.4. I am patient during the mentoring of students.
	II.5. I am supportive of students.
	II.6. I value the student as a member of the health care team.
	II.7. As a mentor, I am fair to all students.
III. Mentor's motivation	III.1. Positive experiences in mentoring students increase my confidence regarding my ability to work as a mentor.
	III.2. Encouragement from colleagues regarding the mentoring of students increases my enthusiasm to mentor students.
	III.3. Constructive feedback regarding my mentoring of students increases my motivation to mentor students.
	III.4. I want to learn and develop as a mentor.
	III.5. I am interested in mentoring students.
IV. Goal-oriented mentoring	IV.1. I guide students in setting the goals that they want to achieve during the clinical practice.
	IV.2. I find out if the student's learning goals are concrete enough so that in practical situations the student knows what his or her goals are and how to attain them.
	IV.3. I find out whether or not the student's learning goals correspond with the learning opportunities provided at the place where the clinical practice is completed.
	IV.4. I clarify to the student what is expected of him or her in order to reach the set goals.
	IV.5. I provide feedback to the student on the goals that he/she has set.
	IV.6. I encourage the student to follow the fulfilment of his or her goals independently.
V. Reflection during mentoring	V.1. During the reflection time, I aim to encourage reciprocal feedback with the student.
	V.2. I try to create a safe atmosphere during the reflection time.
	V.3. I encourage the student to share his or her experiences.
	V.4. I relate empathetically to the student's experiences.
	V.5. I am aware that the student's experiences are unique and significant for his/her learning.
	V.6. I believe that discussion on the student's experiences improves his/her learning.
VI. Student-centred evaluation	VI.1. I encourage the student to remember his/her experiences as they happened and to evaluate them.
	VI.2. During the evaluation, I guide the student in dealing with possible negative feelings.
	VI.3. I ask the student to critically and holistically reflect upon why things happened the way they did.
	VI.4. I encourage the student to evaluate the situation from many perspectives / to find alternative explanations for events.
	VI.5. I emphasise that the evaluation of one's own learning can bring forth new thoughts, feelings and performances that the student may not have previously been aware of.
	VI.6. I guide the student to question what is regarded as self-evident.
	VI.7. I support the student in evaluating his or her own activities.
	VI.8. I encourage students to actively deal with their experiences during the entire clinical practice.
	VI.9. I reflect upon which activities could be developed and how together with the student.
VII. Constructive feedback	VII.1. At the end of the clinical practice, I give a positive final evaluation of the student's performance.
	VII.2. I provide feedback immediately following a certain activity.
	VII.3. I provide feedback for the future and development of the student.
	VII.4. I provide feedback so that the student can change their practices.

In each of the MCI's 43 items, nurse mentors’ level of agreement is scored using a four-point Likert scale: 1 = totally disagree, 2 = disagree to some extent, 3 = agree to some extent and 4 = totally agree. There is no option to select ‘do not know’ or ‘do not want to answer’. There is currently no established method for scoring the MCI. In this research, the participant subscale scores (on a scale of 1–4) were calculated by summing the participant's answers to the items included in the subscale and dividing that sum by the participant's number of answers on the subscale. Higher scores indicate higher levels of agreement.

### 2.4 Translation procedure

Permission to translate, validate and use the English version of the MCI was obtained by e-mail communication between the instrument developer and the last author of this paper (AAGN). The MCI was translated from English into Norwegian following the symmetrical translation approach and seven-step guideline supported by Sousa and Rojjanasrirat (2010), including six of the seven recommended steps: (1) forward translation, (2) forward translation synthesis, (3) backward translation, (4) backward translation synthesis, (5) cognitive debriefing and (7) full psychometric testing. Step 6, preliminary psychometric testing, was not taken due to the impossibility of accessing a bilingual population. Furthermore, that step is rarely applied.

#### 2.4.1 Forward and backward translation and synthesis

The forward translation was done independently by two translators, both of them registered nurses and researchers who were familiar with the terminology of the area covered by the MCI. The translators’ native language was Norwegian, and both were fluent in English. The two forward-translated versions of the instrument by the translators were initially compared with the original version of the instrument by a third bilingual, bicultural independent translator. Identified discrepancies in words, sentences and meanings were resolved by consensus among the translators and the last author (AAGN).

The backward translation was done by two independent back translators who translated the initial Norwegian-translated version of the instrument back into English. One of the back translators was a registered nurse and researcher who was familiar with the terminology of the area covered by the MCI, and the other back translator was an English expert familiar with terms used in the nursing field. The back translators’ native language was English, and both had lived in Norway for more than 30 years. The back translators were completely blinded to the original version of the instrument and there were produced two back-translated versions of the instrument from this process, which were compared by a third bilingual, bicultural independent translator. Identified discrepancies in words, sentences and meanings were resolved by consensus among the involved translators, the last author (AAGN) and the instrument developer who approved the pre-final Norwegian version of the MCI.

#### 2.4.2 Cognitive debriefing

The pre-final Norwegian version of the MCI was tested by a pilot group of 10 registered nurses who had experience of mentoring nursing students in clinical practice and represented the target population (nursing students’ mentors) as recommended (Beaton et al., 2000; Sousa and Rojjanasrirat, 2010). Each registered nurse was asked to rate the MCI—i.e., the instructions, response format and 43 items of the instrument—using a dichotomous scale (clear or unclear). The registered nurses who rated the instructions, response format or any item of the instrument as unclear were asked to provide suggestions on how to rewrite the statements to make the language clearer. The registered nurses did not identify any unclarity, so the instrument was not re-evaluated (Topf, 1986).

Additionally, an expert panel of 10 members was invited to evaluate the conceptual equivalence (clarity) of the instrument (Waltz et al., 2005). The panel comprised experienced nurse mentors and nursing teachers, all of whom were registered nurses and familiar with the terminology of the area covered by the MCI. They followed the same procedures as intended for the pilot group, and the results identified no lack of conceptual equivalence, similar to those described above. The expert panel was asked to evaluate each item of the instrument for content equivalence (relevance) using the following scale: 1 = not relevant, 2 = unable to assess relevance, 3 = relevant, but needs minor alteration and 4 = very relevant and succinct. None of the items were classified as 1 (not relevant), 2 (unable to assess relevance) or 3 (relevant, but needs minor alteration), so there was no need to revise the instrument (Waltz et al., 2005). The instrument was then ready for psychometric testing.

#### 2.4.3 Psychometric testing

##### 2.4.3.1 Sample size and data collection

A convenience sampling was utilized. We aimed for 450 participants to obtain at least 10 responses per item in the instrument, including some insurance in case of withdrawal from the study as recommended by Sousa and Rojjanasrirat (2010). The data were collected using an online survey system (QuestBack). The survey contained an online version of the MCI and an informed consent form developed by AAGN and JZ. This collectively provided data encompassing participants’ name, e-mail and the MCI scores. It is important to note that no additional background data were collected during this study. The participants had to answer all the MCI items to complete the survey so as to avoid missing data.

The online MCI was distributed mainly via an e-mail that included information about the study, a request for participation and a link that directed the participants to the survey. AAGN, KH, MEK and SCWL contacted managers at various nursing homes, hospitals, mental health care units and home nursing care providers in multiple regions of Norway (Oslo, Kristiansand and Tromsø), who then further distributed the e-mail and link to the survey to nurse staff who were eligible participants (registered nurses with experience in mentoring nursing students in clinical practice). The authors also visited health care units, sent e-mails to nurses in clinical placements and networks and recruited face-to-face in networks and in courses for registered nurses enrolled in master's programmes. Up to two reminders were sent by e-mail. The data for psychometric testing were collected digitally from February 2021 through December 2022.

##### 2.4.3.2 Data analysis

The data were analysed using IBM SPSS Statistics version 28 (IBM Corp.) and AMOS Graphics, an IBM SPSS module. For the statistical analysis, we numbered the MCI items (Table 1) from 1 through 43 in sequential order.

We performed CFA using maximum likelihood to investigate whether the hypothesised seven-factor MCI model fit our observed data as evidence of construct validity (Sun, 2005). The fit of the hypothesised model was assessed by the following goodness-of-fit indices: The chi-square value (χ^2^) to degrees of freedom (df) ratio (χ^2^/df ratio), the p-value, the standardised root mean squared residual (SRMR), the comparative fit index (CFI) and the root mean square error of approximation (RMSEA). A χ^2^/df ratio of between 2 and 3 is considered acceptable (Carmines and McIver, 1981). The p-value is used to reject a null hypothesis representing perfect fit (Browne and Cudeck, 1993; Lewis, 2017). Thus, a non-significant p-value is preferred. The acceptable range of the SRMR index is 0.0 to 0.08 (Hu and Bentler, 1999). The CFI ranges between 0 and 1, with higher values indicating a better fit (Sun, 2005); following Bryant and Yarnold (1995), a CFI cut-off point of > 0.90 was deemed acceptable. Lower RMSEA values indicate a better fit (Sun, 2005), and a value of 0.06 or less is considered to represent an acceptable model fit (Schermelleh-Engel et al., 2003).

Regarding internal consistency, Cronbach's alpha values exceeding 0.7 are classified as good (Streiner, 2003). We checked the Cronbach's alpha values, if an item was deleted, to determine whether all the items contributed to the subscales they were assumed to belong to. We also checked that all the items were more highly correlated with the subscale they were assumed to belong to than with any other subscale (corrected item total correlation).

#### 2.4.4 Ethical considerations

The study was approved by the institutional review board at Lovisenberg Diaconal University College and by Norwegian Social Science Data Services (approval no. 803076). We also obtained approval from the health care institutions that demanded approval to collect data. Participation was based on written informed consent, which was a part of the online version of the MCI. The participants were required to read the informed consent, enter their names and provide their e-mail addresses before completing the MCI. This process was implemented to ensure their right to withdraw from participation in the study and request the deletion of their data. By completing these three steps, they agreed to participate in the study as described in the informed consent. The name and e-mail data were anonymised at the beginning of the data analysis process.

## 3 Results

A total of 458 eligible participants completed and returned the survey. The responses were skewed towards the ‘totally agree’ end of the scale, but a full range of responses (1–4) was observed.

### 3.1 Construct validity

**Fig. 1:**
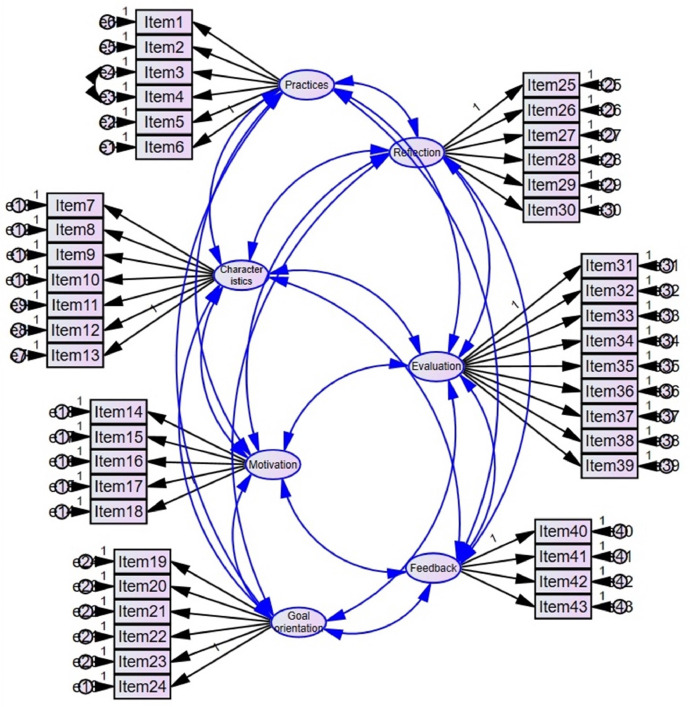
Factor structure model for the MCI (Norwegian version) (*N* = 458).

Fig. 1 shows the factor structure model of the MCI (Norwegian version). The error terms of items 3 and 4 had a modification index of 54.4. Because the wording of the two items appear similar, and correlated errors may arise from items that are similarly worded (Brown, 2015), the error terms of items 3 and 4 were linked. The content of the items is provided in Table 1.

Table 2 presents the CFA goodness-of-fit indices for the factor structure model. Factor loadings within each factor ranged from 0.57 to 0.92 (‘mentoring practices in the workplace’: 0.69–0.80, ‘mentor's characteristics’: 0.72–0.90, ‘mentor's motivation’: 0.69–0.84, ‘goal-oriented mentoring’: 0.72–0.81, ‘reflection during mentoring’: 0.79–0.92, ‘student-centred evaluation’: 0.71–0.80 and ‘constructive feedback’: 0.57–0.81).

**Table 2:** Goodness-of-fit indices from the confirmatory factor analysis (*N* = 458).

χ^2^	df	χ^2^/df	p-value	SRMR	CFI	RMSEA
2134.157	838	2.547	< 0.000	0.051	0.919	0.058

χ^2^ = chi-square; df = degrees of freedom; χ^2^/df = chi-square to degrees of freedom ratio; SRMR = standardised root mean squared residual; CFI = comparative fit index; RMSEA = root mean square error of approximation.

### 3.2 Internal consistency

Table 3 presents the Cronbach's alpha values of each of the seven subscales, which varied between 0.77 and 0.95. The exclusion of any item from its own subscale did not increase the alpha values. Almost all the items (98 %) were most strongly correlated with the subscales as the hypothesised CFA included them. The exceptions were item VII.1. (‘At the end of the clinical practice, I give a positive final evaluation of the student's performance’), which was more strongly correlated with ‘mentor's characteristics’, ‘goal-oriented mentoring’, ‘reflection during mentoring’ and ‘student-centred evaluation’, and item VII.4. (‘I provide feedback so that the student can change their practices’), which was more strongly correlated with ‘student-centred evaluation’.

**Table 3:** Mean score with SD and Cronbach's alpha by subscale (*N* = 458).

Factor/subscale	Mean	SD	Cronbach's alpha (α)	Skewness	Kurtosis
Mentoring practices in the workplace	3.35	0.60	.88	−1.1	1.3
Mentor's characteristics	3.73	0.51	.94	−3.7	15.9
Mentor's motivation	3.62	0.57	.89	−2.5	7.5
Goal-oriented mentoring	3.46	0.57	.90	−1.6	3.6
Reflection during mentoring	3.77	0.52	.95	−3.9	17.4
Student-centred evaluation	3.44	0.56	.92	−1.4	3.0
Constructive feedback	3.43	0.53	.77	−1.5	3.7

SD = standard deviation.

## 4 Discussion

The Norwegian version of the MCI demonstrated acceptability, acceptable construct validity and good internal consistency. To ensure that the conclusions drawn from the statistical analyses were based on differences and similarities between cultures and not on errors in translation, we emphasised equivalence between the original and the translated version of the MCI in the translation process (Wang et al., 2006). We found that it was not difficult to find Norwegian words and expressions that captured the original MCI's meaning. The target population group of nurse mentors who piloted and evaluated the MCI (Norwegian version) confirmed the relevance of the wording and items, indicating the acceptability of the MCI in the Norwegian context.

The construct validity of the MCI (Norwegian version) can be considered acceptable as judged by the CFA's goodness-of-fit indices (Lewis, 2017; Sun, 2005). Mikkonen et al. (2020) used an exploratory factor analysis to develop the original seven-factor MCI and performed CFA to confirm the model fit, resulting in the hypothesised factor model used in our study. We did not redefine the hypothesised factor model, but one minor adjustment was made: the error terms of items I.3. and I.4. were linked, as these items’ wording appeared similar and both contained the possibly ambiguous wording ‘generally agreed practices’, which could be read as ‘generally agreed practices among me and my co-workers’ or as ‘generally agreed practices in official guidelines’ (Table 1). Thus, the error terms in these two items may be related, as both exhibited the same interpretational uncertainty. We evaluated various aspects of goodness-of-fit by using the χ^2^/df ratio, SRMR, CFI and RMSEA fit indices. Our χ^2^/df ratio was within the limit recommended by Carmines and McIver (1981), the SRMR was within Hu and Bentler's (1999) range for acceptable fit, the RMSEA met the criteria suggested by Schermelleh-Engel et al. (2003), and the CFI was above the minimum limit for acceptable fit recommended by Bryant and Yarnold (1995). However, cut-off limits for goodness-of-fit indices are not all universally agreed upon, and Bryant and Yarnold's (1995) CFI cut-off limit for acceptable fit is considered liberal by Hu and Bentler (1999) who argue that a value of 0.95 is needed.

One goodness-of-fit indicator that challenged our model was the significant p-value, which rejected the null hypothesis representing perfect fit. However, models are almost always incorrect to some degree, and this χ^2^ test of exact fit often rejects the null hypothesis representing perfect fit, even when the postulated model is only trivially false (MacCallum, 2003).

The internal consistency of the MCI (Norwegian version) can be considered as good. In the English MCI, the Cronbach's alpha values of all the subscales were between 0.83 and 0.94 (Mikkonen et al., 2020), which is in line with the present study's results ranging from 0.77 to 0.95. The Cronbach's alpha values for all seven hypothesised subscales demonstrated internal consistency, and the exclusion of any item from its own subscale did not increase the alpha values. Moreover, the fact that almost all the items were more strongly correlated with their own subscale than with any of the other subscales confirms that responses were grouped in the manner hypothesised by our model.

An important step in improving nursing students’ clinical education is to ensure that their nurse mentors possess sufficient mentoring competence. Until now, no valid instrument to assess nursing student mentors’ competence in clinical practice was available in Norway. The results of this study demonstrate the MCI's potential as an instrument for assessing current and required competencies of nursing students’ mentors in the Norwegian context. The MCI may be used by nurse mentors in health care institutions and departments that provide clinical practice placements for nursing students in Norway to identify areas of mentoring competence that need to be strengthened.

### 4.1 Strengths and limitations

The main recruiting tool used in this study was e-mail, which made it possible to reach a great number of potential participants while giving the invited nurse mentors the opportunity to respond when appropriate, however the variety of recruiting methods applied makes it difficult to precisely quantify the total outreach and calculate a response rate. Although some of the responding nurse mentors stated that it was an important study, we found that the recruitment went slowly, which is in line with previous research using web-based recruitment (Koo and Skinner, 2005), but we improved the response results by sending e-mail reminders. The suggested minimum size for conducting factor analysis differs in absolute numbers from 100 to over 1000 and in relative terms from 3 to 20 times (3:1–20:1) as many respondents as the number of items (Mundfrom et al., 2005). In this first Norwegian translation and testing of the MCI, the construct validity and internal consistency tests were performed on observed data from a sample of participants that was considered acceptable, as our respondent-to-item ratio was above 10:1 (Bryant and Yarnold, 1995). We did not gather individual characteristics of our participants, such as age, background and years of mentoring experience. Gathering such data could have provided a more comprehensive overview of the sample.

The use of multiple fit indices provided a holistic view of acceptable goodness-of-fit (Sun, 2005). However, because the cut-off values for fit indices are not all universally agreed upon, caution should be taken against strict reliance on the selected cut-offs (Hu and Bentler, 1999; Sun, 2005). Moreover, we did not evaluate the MCI's stability over time. We therefore recommend that future research confirm the factor structure of the MCI (Norwegian version) and include evaluations of the instrument's test-retest reliability.

## 5 Conclusions

The MCI (Norwegian version) demonstrated its potential as a useful instrument to assess current and required competencies of nursing students’ mentors in clinical practice in nursing education. It is important to gather data in these areas to inform the development, evaluation and enhancement of mentorship competence. The MCI (Norwegian version) exhibited good internal consistency and acceptable construct validity in this study, but future research should confirm the factor structure of the MCI (Norwegian version) and evaluate its test-retest reliability.

## Funding

This research did not receive any specific grant from funding agencies in the public, commercial, or not-for-profit sectors.

## CRediT authorship contribution statement

**Silje Christin Wang Linnerud:** Data curation, Investigation, Project administration, Visualization, Writing – original draft, Writing – review & editing. **Camilla Olaussen:** Data curation, Formal analysis, Visualization, Writing – original draft, Writing – review & editing. **Jaroslav Zlamal:** Conceptualization, Data curation, Investigation, Writing – original draft, Writing – review & editing. **Monica Evelyn Kvande:** Investigation, Writing – review & editing. **Kristine Haddeland:** Investigation, Writing – review & editing. **Andréa Aparecida Goncalves Nes:** Conceptualization, Investigation, Supervision, Visualization, Writing – original draft, Writing – review & editing.

## Declaration of competing interest

We have no conflicts of interest to disclose.
